# A rhombus–keyhole shaped circularly polarized dual-band implantable antenna with low SAR compliance

**DOI:** 10.1371/journal.pone.0354130

**Published:** 2026-07-21

**Authors:** Nagarajan Vasugi, Madurakavi Karthikeyan

**Affiliations:** Department of Communication Engineering, School of Electronics Engineering, Vellore Institute of Technology, Vellore, Tamil Nadu, India; Parul University, INDIA

## Abstract

A compact dual-resonance implantable antenna with circular polarization (CP) has been proposed in this paper for bio telemetry applications. The antenna, with dimensions of $\text{9}\;\times \;\text{9}\;\times \;\text{1}.\text{2}\text{7}\;{mm}^{\text{3}}$, is designed using a reversed L-shaped patch, dual key-hole shaped structures and a central rhombus-shaped element. A shoring pin is employed to achieve dual-band resonance, while structural modification enables circular polarization. The proposed antenna resonates at 1.4 GHz (WMTS band) and 2.45 GHz (ISM band). The antenna is fabricated and measured using minced pork meat and saline solution. The measurement results show good agreement with the simulation results. Further, the SAR analysis and link budget analysis, prove that the antenna is suitable for real-time applications.

## 1. Introduction

With the rapid growth of recent services for health care technologies, implantable medical devices (IMDs) are essential for patients’ diagnosis and care [1]. These implantable medical devices are used in different medical applications, including cardiac leadless pacemakers [2], retinal prosthesis [3], skin application [4], continuous blood sugar monitoring [5], capsule endoscopy [6], brain-machine interface and intracranial pressure monitors [7]. The biological data are wirelessly transferred to the external world; the antenna plays a crucial role in IMD’s [8]. However, we must take into account a few limitations while constructing the implantable antenna, including miniaturization, bio-compatibility, improved efficiency, impedance matching, patient safety, and work in the medical frequency band. The implantable medical devices operate at different frequency range, which is allocated by Federal Communication Commission (FCC), such as WMTS Bands (1395−1400 MHz, 0.608–0.614 GHz) MICS (0.402–0.405 GHz), ISM (0.43305–0.43479 GHz, 0.902–0.928 GHz, 2.38–2.4835 GHz and 5.725–5.850 GHz) Med Radio (0.401–0.406 GHz, 0.413–0.419 GHz, 0.451–0.457 GHz 0.426–0.432 GHz and 0.438–0.444 GHz) [9]. Among this medical frequency band, the WMTS and ISM are used for bio-telemetry remote health monitoring and wireless data transmission applications.

The frequent activities and postural movements of the human body lead to the multipath reflection. Due to this, the polarization mismatch will occur in the linear polarization (LP) antenna [10]. An antenna with circular polarization (CP) is a good option for dependable communication [11,12]. In [13], by using two triangular radiators embedded at the bottom and top of the proposed antenna, exhibiting 2.45 GHz with a size of $\text{9}.\text{2}\;\times \;\text{9}\;.\text{2}\times \;\text{1}.\text{27 }{\text{mm}}^{\text{3}}$. Additionally, the ARBW of 3.7% was attained through V-shaped slots in the patch. The shorting pin and quarter-wavelength inverted- F shaped structure of the reported antenna achieved miniaturized and dual-band resonance at WMTS and ISM bands. Additionally, at the lower band, the circular polarization was achieved by using two crossed strips, placed orthogonally, as reported in [14]. Nevertheless, the SAR is high, the antenna size is large, and its gain and ARBW are low. With a size of $\text{9}.\text{8}\;\times \;\text{9}\;.\text{8}\times \;\text{1}.\text{27 }{\text{mm}}^{\text{3}}$, the antenna used a notched ring slot in the patch to operate at the 2.4 GHz band. Additionally, the CP was exhibited at the same band with ARBW of 15.8% through creating two C-shaped slots in the radiating element. However, ARBW is low, and an antenna having a high volume with low gain has been reported in [15]. In [16], by using a horizontal U-shaped patch and a shorting pin, the antenna achieved dual resonance with the physical size of $\text{9}\;\times \;\text{9}\;\times \;\text{1}.\text{27 }{\text{mm}}^{\text{3}}$, operating at UHF and ISM-band. Additionally, circular polarization was achieved by using DGS. However, the SAR value is high, and the axial ratio bandwidth is low. In [17], the antenna exhibits dual resonance and dual-circular polarization by using characteristic mode analysis. Even though dual-CP was achieved with less volume of proposed antenna, the antenna reports a high SAR value and a narrow ARBW. In [18], the compact implantable duplex antenna with an open-end slot, a semi-circular slot, and a shorting pin is proposed for data telemetry at 2.4 GHz and wireless power transfer at 1.8 GHz. Employing an in-band full duplex antenna function at 2.45 GHz ISM band with a compact volume of 7.17 mm^3^, is used for capsule endoscopy application, has been reported in [19].

From the above studies, it is observed that achieving a compact implantable antenna with wide axial ratio bandwidth, low SAR, and stable circular polarization at dual bands remains a significant challenge. Most existing designs suffer from trade-offs between size, ARBW, SAR, and gain, particularly for WMTS and ISM band applications. This forms the key research gap addressed in this work.

Therefore, the main objective of this work is to design a compact dual-band implantable antenna that simultaneously achieves (i) circular polarization with wide ARBW, (ii) operation in WMTS (1.4 GHz) and ISM (2.45 GHz) bands, (iii) low SAR within IEEE safety limits, and (iv) improved impedance matching while maintaining a compact size.

A compact, dual resonant implantable antenna with circular polarization is proposed in this article for use in bio-telemetry applications. With a low SAR value, the design works at two operating bands (2.45 GHz ISM band and 1.4 GHz WMTS band) and achieved a broad ARBW of 33.2% at the ISM band, with a compact size of $\text{9}\;\times \;\text{9}\;\times \;\text{1}.\text{2}\text{7}\;{mm}^{\text{3}}$. The center rhombus-shaped, reversed L-shaped patch and shorting pin of the designed antenna help to achieve a compact size and dual-band. The rectangular patch added between the rhombus and circular-shaped patch helps to achieve better impedance matching. Additionally, by using the keyhole structure-shaped patch, the antenna exhibits CP at the ISM band. The antenna achieved LP/CP property at 1.4/2.45 GHz. The CST Microwave studio suite is used to design and simulate the antenna performance. The designed antenna has been tested by embedding the antenna out of the top at 4 mm into a homogeneous skin phantom. Minced pork meat and skin-mimicking solution were used to evaluate the simulated outcomes.

This paper’s remaining content is laid out as follows. In Section 2, geometry layout, parametric analysis, evolution stages and CP mechanism. In Section 3, measured and simulated results are reported. Conclusion provided in Section 4.

## 2. Methodology of the designed antenna

### 2.1. Antenna design and geometry layout

**Fig 1:**
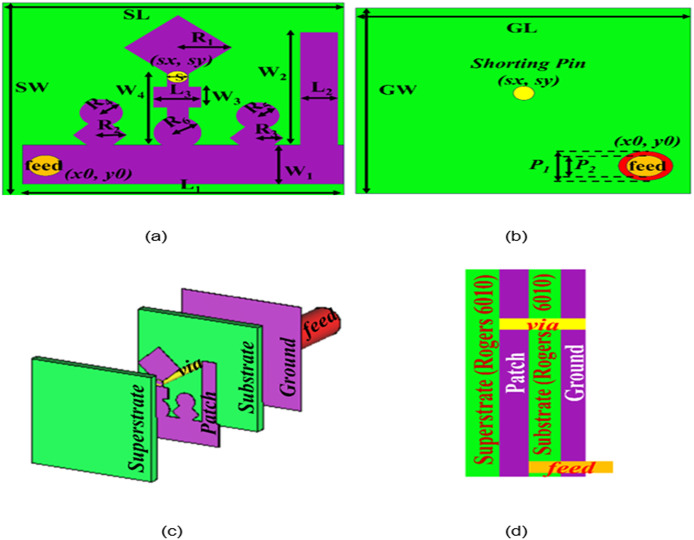
Layout of the designed antenna (a) Radiating element, (b) Ground plane, (c) Exploded view and (d) lateral view.

Fig 1a-1d depicts the designed antenna’s precise geometric arrangement. The antenna radiated element is composed of a reversed L-shaped, circular-shaped, Rhombus-shaped, two-keyhole structure patch and shorting pin, as shown in Fig 1(a). In Fig 1(b), the ground plane is displayed. The exploded profile present in Fig 1(c) and the lateral profile of the antenna present in Fig 1(d). The Rogers 6010, a biocompatible substrate material with ℰr = 10.2 and tan δ = 0.0023 is used for constructing the antenna with a thickness of 0.635 mm. To prevent human tissue from damage, the same biocompatibility substrate material is used as a superstrate. The reversed L-shaped patch and shorting pin helped to exhibit dual-band resonance. Additionally, the center circular and rectangular-shaped patches help in achieving the impedance matching of lower and higher resonance bands. The two-keyhole structure attached to the left and right sides of the reversed L-shaped patch helps to attain CP at a higher band. The dimension of the antenna is $9\;\times \;9\;\times \;1.27\;{𝐦𝐦}^{3}$ (in terms of $0.0420\;\lambda_{0}\;\times 0.0420\;\lambda_{0}\;\times 0.0059\;\lambda_{0},$ at the lower band where $\lambda_{0}\;$is calculates). The optimized antenna dimensions are listed in Table 1.

**Table 1:** Optimized variable value.

Symbol	Value (mm)	Symbol	Value (mm)
SL, SW, GL, GW	9	W_1_	2
L_1_	8.5	W_4_	3.5
W_2_	5.5	L_2_	1.05
L_4_, S	0.6	R_2_, W_3_, R_3_	1
L_3_	1.4	R_4_, R_5_	0.7
R_1_	1.6	R_6_	0.8
P_2_	1.2	P_1_	0.6
(sx, sy)	(0, 0.5)	(x0, y0)	(−3.25, −3.25)

### 2.2. Design and evolution stages

**Fig 2:**
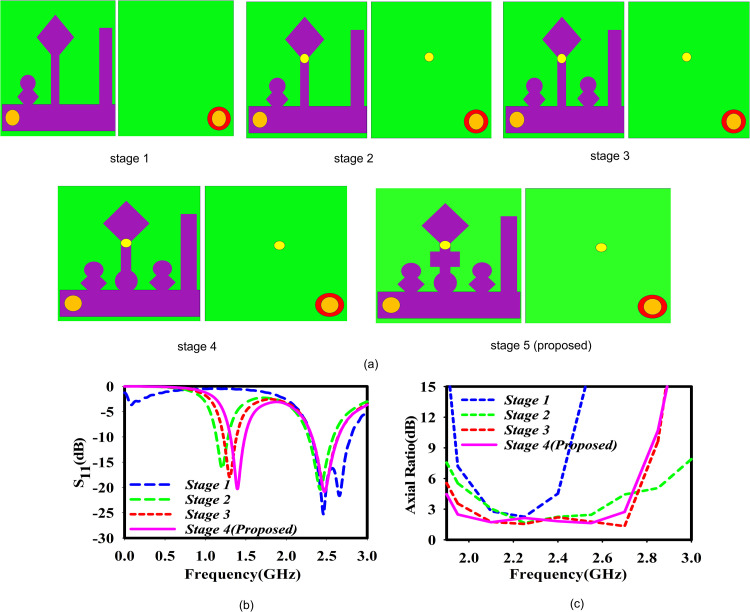
(a) Evolution stages, (b) Comparison of S11 of each stage, and (c) Comparison of AR of each stage.

The complete steps involved in the process of designing the antenna are laid down in Fig 2(a). The reflection coefficient (S_11_) and AR of each step are depicted in Fig 2(b) and 2(c), respectively. The design process begins with reversed L-shaped, center Rhombus-shaped, and keyhole-shaped patches with a complete ground structure. As a result, the radiating element with S_11_ of −17 dB at 2.3 GHz is achieved. However, at this stage, circular polarization (CP) is not attained. We introduced a shorting pin at (Sx, Sy) with the full ground in stage 2. Through this modification, the design produced the dual-band resonance S_11_ of −16.2 dB at 1.2 GHz and S_11_ of −20.5 dB at 2.4 GHz. However, the required orthogonal modes for CP are not sufficiently excited, and hence CP is not achieved as shown in Fig 2(c). The mirror of the keyhole-structured patch is added on the right side of the bottom of the reversed L-shaped patch in stage 3. Then, the second band is shifted from 2.4 GHz to 2.42 GHz with an S_11_ of −21.5 dB, and the lower band is unchanged. This structural asymmetry enables the excitation of two near-degenerate orthogonal modes with improved current distribution along perpendicular directions. As a result, CP is achieved at the higher band (2.42 GHz), where the axial ratio falls below 3 dB, as shown in Fig 2(c). Even though the CP is achieved at the higher band, the lower band is not our targeted band. Moreover, a very narrow ARBW is observed at this stage. The circular-shaped radiating element is added at the bottom of the center arm of the patch at stage 4. This addition enhances mode coupling and improves the phase quadrature between orthogonal current components. Due to this, the lower band shifted to 1.33 GHz from 1.2 GHz, and the higher band moved within the ISM band range at 2.45 GHz with an axial ratio of 2.1 dB. In stage 5, in the center of the rhombus arm patch, a rectangular-shaped patch is added. This further refines the current distribution and stabilizes the orthogonal modes, resulting in improved impedance matching and wider AR bandwidth. At this stage, our designed antenna achieved a targeted at 1.4 GHz WMTS band with S_11_ of −20.51 dB and 2.45 GHz with −20.70 dB S_11_ with a wide ARBW of (1.935–2.705) 33.2% as shown in Fig 2(c). The center circular-shaped patch helps to fix impedance matching of the higher band, and the keyhole-shaped patch helps to achieve CP at the higher band. The center rectangular-shaped patch helps to fix impedance matching of the lower band frequency.

### 2.3. Parametric analysis of the proposed antenna

In this section, we provide extensive parametric analysis of key elements, including W_1_, L_2_, and the shorting pin of the designed antenna to understand the impact of various antenna elements.

#### 2.3.1. Variation in W_1_.

**Fig 3:**
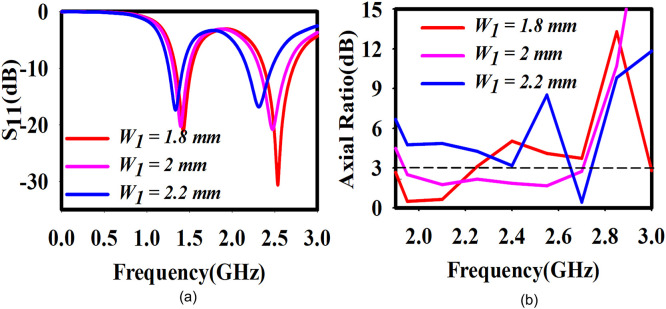
Varying W_1_ on (a) S_11_ and (b) AR.

As illustrated in Fig 3(a) and 3(b), the antenna width W_1_ is adjusted from 1.8 mm to 2.2 mm. Due to these adjustments of W_1_, the S_11_ and AR are significantly affected. Initially, W_1_ was set to 2 mm. When we adjust W_1_ = 1.8 mm, the lower and upper band S_11_ values slightly shift to the right side of the targeted band, while the AR in the upper band is significantly affected. When we adjust W_1_ = 2.2 mm, the S_11_ of the lower band and upper band are significantly affected, and AR is also dramatically affected. When W_1 is_ set to 2 mm, both bands of S_11_ resonate at the targeted WMTS and ISM bands. At the same time, AR in the upper band is less than 3 dB with a wide ARBW of 770 MHz. Hence, the W_1_ is the crucial element for impedance matching and the CP property of the proposed design.

#### 2.3.2. Variation in L_2_.

**Fig 4:**
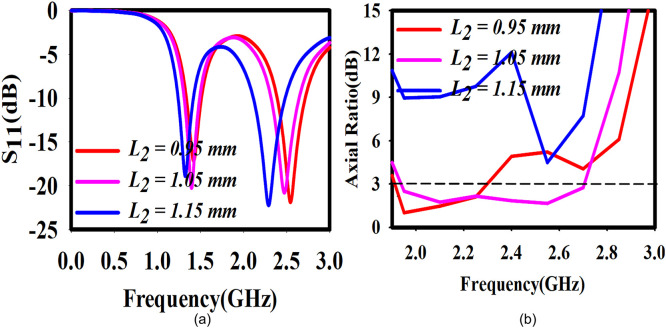
Effect of varying L_2_ on (a) S_11_ and (b) AR.

The length L_2_ plays a dominant role in achieving a dual desired band resonance and circular polarization property. Fig 4(a) and 4(b), we can clearly observe that when we set L_2_ = 1.15 mm, the resonance of the higher and lower bands changes drastically. The respective AR of the upper band is less than 3dB. When L_2_ = 0.95 mm, the resonance slightly moves towards the right at 1.45 GHz (with S_11_ = −16 dB) and 2.6 GHz (with S_11_ = −21 dB). The corresponding AR is greater than 3 dB. When we set L_2_ = 1.05 mm, the antenna achieved the desired dual-targeted band at 1.4 GHz and 2.45 GHz. The respective AR is < 3 dB at 2.45 GHz with ARBW of 33.2%.

#### 2.3.3. Variation in the location of the shorting pin.

**Fig 5:**
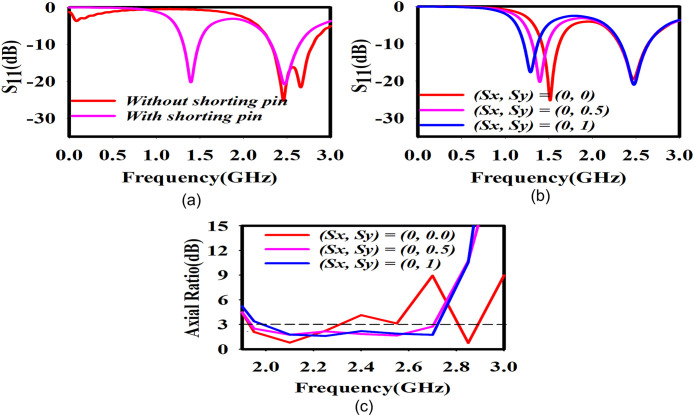
(a) S_11_ with and without shorting pin. Effect of varying location of shorting pin on (b) S_11_ and (c) AR.

To achieve dual-band resonance of the designed antenna, the shorting pin location plays a vital on dual-band resonance. From Fig 5(a), we can clearly observe that without the shorting pin, the antenna exhibits a single band with S_11_ of – 29 dB at the higher band. After inserting the shorting pin, the designed antenna exhibits dual-band resonance. Hence, the analysis was performed over the location of the shorting pin (*Sx, Sy*). The location of shorting pin is varied as *Sx = 0, Sy = 0*, *Sx = 0, Sy = 0.5* and *Sx = 0, Sy = 1*. When we choose *Sx = 0, Sy = 0* and *Sx = 0, Sy = 1*, mainly affects S_11_ of the lower band while the upper band is not affected. When we set *Sx = 0, Sy = 0.5,* the antenna resonates at 1.4 GHz and 2.45 GHz with good impedance aligned as displayed in Fig 5(b). The respective circular polarization is attained at 2.45 GHz as shown in Fig 5(c).

### 2.4. CP Mechanism of implantable antenna

**Fig 6:**
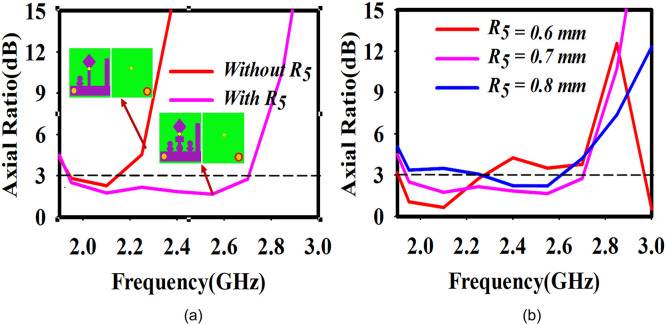
Variation of CP (a) with and without keyhole structure (b) Effect of varying R_5_ on AR.

Fig 6(a) reveals the circular polarization mechanism in this design. Without adding the keyhole structure, the antenna has not achieved the CP property at any band of the design. After adding a keyhole structure in right side of the reversed L-shaped patch, the antenna exhibits < 3dB AR at the upper band as described in Section 2.2, stage 3, Fig 2(c). Therefore, we investigated the keyhole structure radius R_5_. As illustrated in Fig 6(b), changing R_5_ to 0.6, 0.7 and 0.8 mm, we observe that R_5_ = 0.6 mm, the AR is affected dramatically, and the AR value is 4.5 dB at the ISM band. The AR value is shifted to 2.2 dB from 4.5 dB at the ISM band. With a narrow AR bandwidth, R_5_ = 0.8 mm. Finally, R_5_ adjusted to 0.7 mm, the ISM band becomes 1.7 dB with a wide AR bandwidth of 33.2% (1.935–2.705 GHz).

## 3. Results and discussion

**Fig 7:**
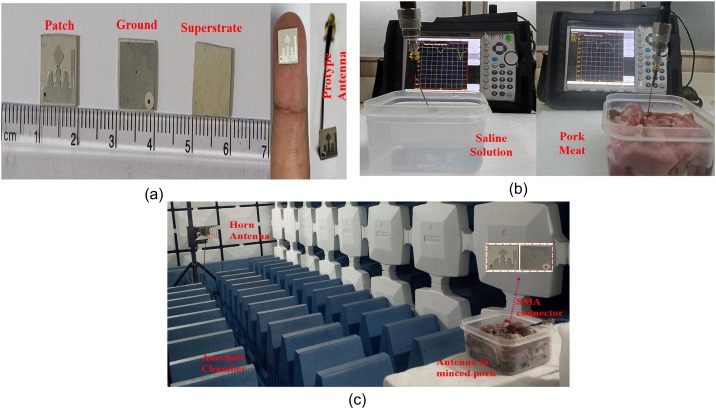
(a) Fabricated prototype antenna (b) S11 measurement setup and (c) far-field test in anechoic chamber.

The prototype antenna was fabricated and measured to confirm the results of the simulation as illustrated in Fig 7a-7c. Initially, S-parameters are tested by using a VNA as shown in Fig 7(b). Then, the test was conducted in an anechoic chamber to test the far-field results present in Fig 6(c). Fig 7(a) illustrates the fabricated antenna.

### 3.1. SAR calculation of designed implantable antenna

**Fig 8:**
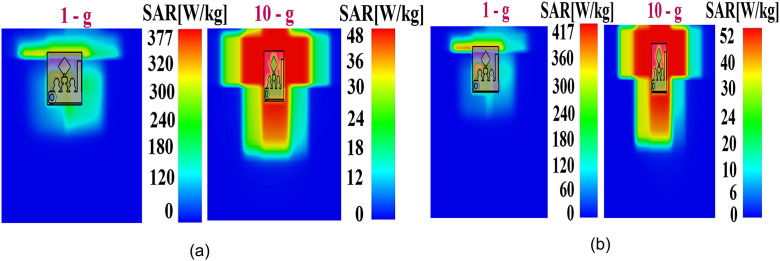
The simulated SAR with a phantom at (a) 1.4 GHz and (b) 2.45 GHz.

Human safety needs to be thoroughly examined because the antenna must operate inside human bodies. So, it is essential to evaluate a SAR analysis to ensure that the antenna complies with IEEE safety guidelines set by the ICNIRP and FCC [20]. The 1-g SAR of human tissue is restricted to 1.6 W/Kg [21] and the SAR of 10-g human tissue is restricted to 2 W/Kg [22] at 1W input power. Fig 8(a) shows the maximum simulated SAR value found over 1-g is 377 W/Kg with the highest permitted power (HPP) of 4.2 mW, and 48.1 W/Kg is found over 10-g with HPP of 41.5 mW at the lower band. At 2.45 GHz, the maximum simulated SAR found over 1-g is 417 W/kg with HPP of 3.8 mW, and 52.6 W/Kg is found over 10-gram with HPP of 38.0 mW at a higher band as shown in Fig 8(b). The antenna conforms with safety standards, as indicated by the SAR and HPP values presented in Table 2. It shows that the designed antenna operates well within the allowable bounds and conforms to the IEEE safety standard when scaled to the standard 25µW [23,24] input power used in a medical implantable antenna.

**Table 2:** SAR AND HPP.

Bio Tissue	Resonant Frequency	SAR (W/kg)		HPP (mW)	
		1-gram	10- gram	1- gram	10- gram
Skin	1.4 GHz	377	48.1	4.2	41.5
	2.45 GHz	417	52.6	3.8	38.0

### 3.2. Surface current of designed implantable antenna

**Fig 9:**
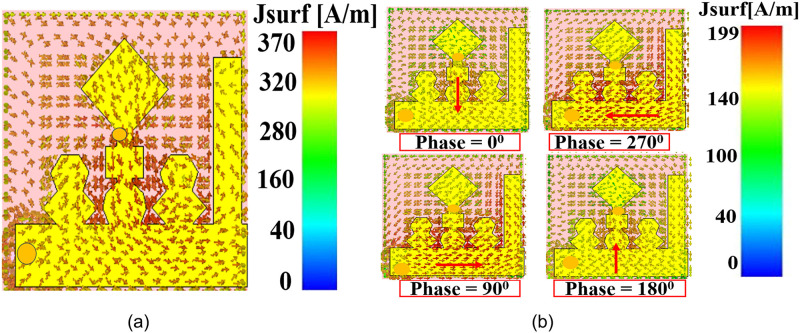
Simulated surface current distribution on radiating element at (a) 1.4 GHz and (b) 2.45 GHz.

In order to have a better knowledge of the functioning of the polarization process at both operating bands, the behaviour of the implantable antennas was investigated by observing the current flow on the antenna’s area in different phases. Fig 9(a) shows the current flow mainly distributed at the bottom of the patch, near the shorting pin and the maximum current flow is observed at the center of the rectangular and circular patch of the lower band. At 2.45 GHz, the antenna is excited at a phase of ${\text{0}}^{\text{0}}$, ${\text{9}\text{0}}^{\text{0}}$, ${\text{1}\text{8}\text{0}}^{\text{0}}$ and ${\text{2}\text{7}\text{0}}^{\text{0}}$ degrees in Fig 9(b). When the phase is excited between ${\text{0}}^{\text{0}}$ and ${\text{1}\text{8}\text{0}}^{\text{0}}$ degree, the entire patch experiences uniform current flow, whereas the maximum current flow in the reversed L- shaped patch, area near the shorting pin, the two-key hole patch and center of the circular patch is experienced when the phase θ is excited between ${\text{9}\text{0}}^{\text{0}}$ and ${\text{2}\text{7}\text{0}}^{\text{0}}$ degrees. We can clearly observe that the main surface current rotating counter clock wise, which indicates the designed antenna exhibits right- hand circularly polarized (RHCP).

### 3.3. Equivalent circuit model

**Fig 10:**
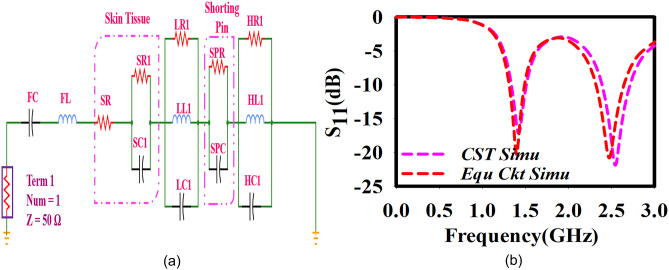
(a) Equivalent circuit model of the implantable antenna using lumped elements (b) Simulated S_11_-parameters using CST and ADS.

The lumped element-based equivalent circuit model of the designed antenna is modelled using ADS software as shown in Fig 10(a). By using an array of RLC-based lumped element circuits, characteristics of antenna dual-band resonance are modelled. The element FC and FL values have been matched with a 50-ohm coaxial cable feeding line [25]. Whereas the SR, SR1 and SC1 represent the skin tissue model [26]. The left side, parallelly connected RLC (LR1, LL1, and LC1), represents the Reversed L-shaped and center rhombus-shaped arm patch, which is dominant in achieving lower band operating frequency. Whereas the higher band operating frequency controlled by the right side, parallelly connected RLC (HR1, HL1, and HC1) depends on the keyhole structure, centrally connected circle and rectangular-shaped patch. The parallelly connected element SPR and SPC between two RLC tank circuit models, the shorting pin [27]. The impedance matching is controlled by two parallel RLC tank circuits. The optimized design is presented in Fig 10(a). The final optimized value is listed in Table 3. There was a noticeable similarity between curves as shown in Fig 10(b).

**Table 3:** Optimized parameter value of an implantable antenna using a lumped element.

*FC (pF)*	*FL (nH)*	*SR (Ω)*	*SR1 (Ω)*	*SC1 (pF)*	*LR1 (Ω)*	*LL1 (nH)*	*LC1 (pF)*	*HR2 (Ω)*	*HL2 (nH)*	*HC2 (pF)*	*SPR (Ω)*	*SPC (pF)*
***999.9***	***15.15***	***7.36***	***500***	***500***	***1000***	***3.48***	***4.495***	***970.85***	***2.48***	***2.04***	***6***	***1.131***

**Fig 11:**
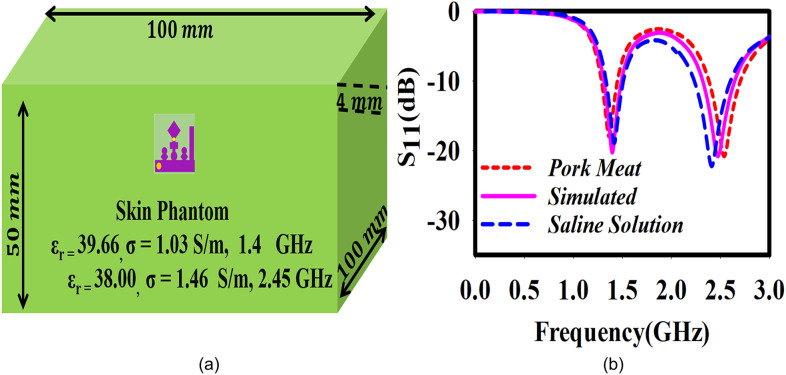
(a) Antenna placed inside the homogenous skin phantom and (b) Comparison of the reflection coefficient simulated and measured results.

The implantable antenna design procedure differs from a traditional free-space antenna. The implantable antenna should be embedded in the human tissues. The homogeneous skin phantom model chosen in CST microwave studio is used to design and analyse the performance of the antenna at 4 mm depth [16,28,29] in a simulation environment. It mimics a realistic human tissue property [14,30,31] and minced pork meat is used to confirm outcomes of the simulation. Fig 11(b) illustrates that the experimental findings demonstrated that the S-parameter values in the two scenarios are comparable.

**Fig 12:**
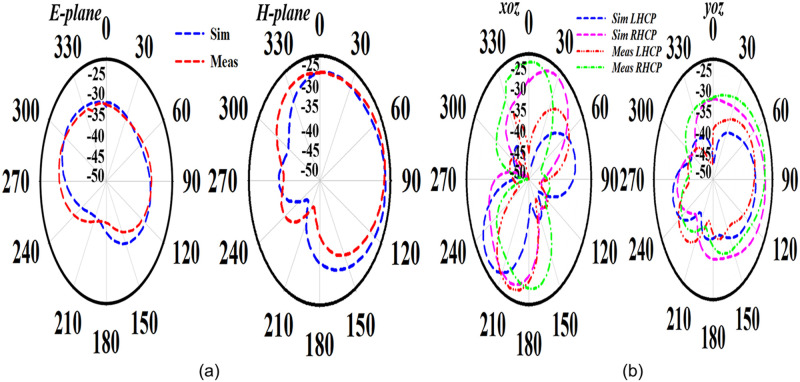
Radiation pattern at (a) 1.4 GHz and (b) 2.45 GHz.

The radiation performance of the proposed implantable antenna was evaluated at both resonant frequencies to validate its polarization behavior and overall suitability for biomedical telemetry. The observed and simulated E-plane and H-plane patterns, which demonstrate good consistency and robust directional properties inside the tissue-emulating environment, support the antenna’s linear polarization within the lower operating band (1.4 GHz) as shown in Fig 12(a). At the higher operating band (2.45 GHz), the antenna demonstrates right-hand circular polarization (RHCP). Effective circular polarization creation is confirmed by the simulated and measured LHCP/RHCP radiation patterns in the xoz and yoz planes, where the RHCP component predominates over the LHCP as shown in Fig 12(b). The circular polarization is attributed to the integrated keyhole-shaped perturbation and the overall patch geometry, which promote the required orthogonal field components with a 90° phase shift. The resilience of the antenna design when placed inside biological tissue is further confirmed by the strong consistency between simulated and measured patterns across both resonant frequencies.

### 3.4. Link budget analysis

The main function of an implantable antenna is to pass data like pictures and videos from inside the human body to the external world. To ensure reliable wireless communication between them, the link margin (LM) analysis can be done. The gain (TGt) of −29/-28.66 dBi acts as the transmitting antenna gain, with an allowable input power of 25µm [2], and receiving antenna gain (RGr) of 2.15 dBi [23,32]. The LM is defined as the difference between the required power (RPR) and the available power (APR). The LM can be analyzed by the following equation [25].


$$\begin{array}{c} \text{LMR}(\text{dB})=(\text{APR}-\text{RPR}), \end{array}$$ (1)

$$\begin{array}{c} \text{APR}(\text{dB})=\text{PRT}+\text{TGt}-\text{ N0}+\text{RGr}-\text{Lfs}, \end{array}$$ (2)

$$\begin{array}{c} \text{RPR}(\text{dB})=\frac{\text{Eb}}{\text{N0}}+\text{10log10}(\text{Br})-\text{TGc}-\text{TGd}, \end{array}$$ (3)

$$\begin{array}{c} \text{Lfs}={\left(\frac{\text{4}\pi \text{d}}{\lambda }\right)}^{\text{2}}, \end{array}$$ (4)

**Fig 13:**
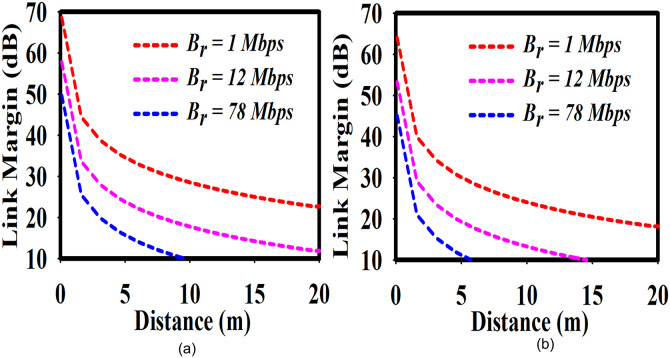
LM analysis at various data rates at (a) 1.4 GHz and (b) 2.45 GHz.

Where, TGc code gain, *d* generally represents the distance between the receiver and transmitter antenna. TGd denotes fixing deterioration, and *Lf*s is the path loss in free space. A 20 dB link margin is fixed [2] to calculate the reliable wireless communication. In this study, the analysis is carried out for three different bit rates (Br) of 1 Mbps,12 Mbps and 78 Mbps at both the operating frequencies. When the data rate is 78 Mbps, the implantable antenna covers a radius of 5 meters at 1.4 GHz band and 3 meters at the 2.45 GHz band. Fig 13(a) and 13(b), we can clearly observe that, at data rates of 12 Mbps and 1 Mbps, the implantable antenna covers a distance of more than 10 meters at both the operating frequencies. Hence, the implantable antenna is a promising option for applications involving biomedical telemetry.

In Table 4, the comparison of the designed antenna with recently published works is given. In [13,15] the designed antenna produced a single-band resonance with circular polarization. However, they possess high volume, high SAR and low gain. However, our designed antenna produced comparably lower SAR and high gain with a compact antenna size. In [17], the author produced a dual-band, dual CP with less antenna volume. However, the design produced low gain with a high SAR value. In [14,16], the authors achieved a dual-band resonance with LP/CP property, having limitations of high antenna volume, high SAR and low gain. However, our designed antenna exhibits dual-band with LP/CP property and produces low SAR, high gain with compact dimensions. Overall, the comparison of our designed antenna depicts that the proposed design achieves dual-band LP/CP property with wide ARBW.

**Table 4:** Comparison with recently published literature.

Ref	Frequency (GHz)	Size *mm*^3^	Volume *mm*^3^	Single/ Dual band	Gain (dBi)	CP YES/ NO	ARBW (<3dB) %	SAR (W/K g)	
								1-g	10-g
**This Work**	**1.4**	9 × 9× 1.27	**102.87**	**Dual**	**−29**	**----**	**---**	**377**	**48.1**
	**2.45**				**−28.66**	**CP**	**33.2**	**417**	**52.6**
[13]	2.45	9.2 × 9.2 × 1.27	107	Single	−24.8	CP	3.7	---	81.5
[14]	1.4	$\pi {\times \text{5}.\text{1}}^{\text{2}}\times \text{1}.\text{2}\text{7}$	103.7	Dual	−32	CP	10.38	692	85.6
	2.45				−31.6	---	----	786.9	90.9
[15]	2.4	9.8 × 9.8 × 1.27	121.97	Single	−33	CP	15.8	486	90
[16]	0.952	9 ×9 × 1.27	102.87	Dual	−27.5	---	---	463	56.9
	2.4				−31.3	CP	20.00	461	55.8
[17]	0.915	$\pi {\times \text{4}.\text{7}}^{\text{2}}\times \text{0}.\text{8}\text{8}\text{9}$	61.66	Dual	−29.5	CP	22.40	585.1	---
	2.45				−19.5	CP	9.48	462.2	---

## 4. Conclusion

This article reports a compact, circularly polarized dual resonance implantable antenna working at two different frequencies, on 1.4 GHz and 2.45 GHz, for applications involving bio-telemetry, with a compact size of $\text{9}\;\times \;\text{9}\;\times \;\text{1}.\text{2}\text{7}\;{mm}^{\text{3}}$. The compactness and dual resonance are attained through, reversed L-shaped patches, shoring pin, center rhombus and two-key hole structure shaped patches. Additionally, the circular polarization is achieved at a higher band with a wide ARBW of 33.2%. Also, the realized peak gain is observed −28.66 dBi. The fabricated antenna was tested in order to verify the simulated outcomes by using minced pork meat and saline solution. The validated results are well aligned with the simulated ones. Furthermore, SAR values for the implantable antenna were simulated, and the results are compiled with the IEEE safety guidelines. The LM analysis was conducted to ensure dependable communication, and the results show that the developed antenna is an optimal choice for applications involving wireless biomedical telemetry.
