# Supplementary material for: A rhombus–keyhole shaped circularly polarized dual-band implantable antenna with low SAR compliance
Source: PLoS One. 2026 Jul 21;21(7):e0354130. doi: 10.1371/journal.pone.0354130 (PMC13387530; doi:10.1371/journal.pone.0354130)
Supplement: Attachment — Submitted filename: Plos one Comment and Response.docx [file pone.0354130.s002.docx]

**Dear editor and reviewers,**

Thank you for your valuable time and comments towards improving the quality of this paper. We have carefully addressed all of your queries. We welcome further suggestions to improve the quality of paper, if necessary.

**REVIEWER REPORT(S):
Reviewer #1:
COMMENTS TO THE AUTHOR(S)**

The authors have designed an implantable antenna with circular polarization. I have the following comments:

**1. More details should be provided on how circular polarization (CP) was achieved. Currently, the explanation lacks sufficient technical depth.**

We thank the reviewer for this valuable comment. We agree that the mechanism of circular polarization (CP) generation was not sufficiently elaborated in the original manuscript. In the revised version, we have clarified the CP generation mechanism by explicitly explaining how orthogonal current modes with a 90° phase difference are introduced through geometrical perturbations and structural asymmetry.

Specifically, the introduction of the shorting pin, mirrored keyhole structure, and additional radiating elements progressively perturb the surface current distribution, enabling the excitation of two near-degenerate orthogonal modes. These modes exhibit the required quadrature phase relationship, leading to circular polarization. The evolution of axial ratio (AR) across stages has also been better interpreted in terms of current distribution and mode coupling

**2. Please provide more details on the location of the shorting pin and explain how its position was chosen.**

We thank the reviewer for this important comment. We gave details on the location of the shorting pin and position of shorting under the section of 2.3.3 in the revised manuscript.

**3. The state of the art should be improved by including more relevant and recent papers, such as: (a) 10.1109/OJAP.2025.3619468 and (b) 10.1109/TAP.2024.3499367.**

We thank the reviewer for valuable suggestion. We have referred the mentioned article in the revised manuscript.

**4. Why was an implantation depth of 4 mm selected? Please justify this choice.**

We sincerely thanks for the reviewer for this insightful comment. The choice of a 4 mm implantation depth was based on the typical subcutaneous implant parameters that are reported in the literature [28], [29], and [16]. Antenna operates in a lossy tissue environment; this depth ensure that the antenna observes actual detuning and absorption effects without encountering the high attenuation caused by deeper implantation. As a results, it offers a realistic and comparable environment for assessing antenna performance.

**5. What is the reason for assuming a 2.15 dB gain for the dipole antenna?**

We thank the reviewer for this important comment. The receiver antenna gain is assumed 2.15 dBi considering that the theoretical gain of a dipole antenna is frequently used as the standard reference in the calculation of link budget analysis. This gain value is often used in the literature to optimize analysis and ensure antenna performance evaluation [23], and [32]. Therefore, this gain value gives a realistic and dependable standard for estimating the link margin.

**Dear editor and reviewers,**

Thank you for your valuable time and comments towards improving the quality of this paper. We have carefully addressed all of your queries. We welcome further suggestions to improve the quality of paper, if necessary.

**Reviewer #2:**

**COMMENTS TO THE AUTHOR(S)**

The candidate has submitted the manuscript titled “A Rhombus–Keyhole Shaped Circularly Polarized Dual-Band Implantable Antenna with Low SAR Compliance”.

The manuscript should be further processed only after the following corrections.

**• The abstract should be a point summary. Unnecessary sentences shall be avoided**

We thank the reviewer for this suggestion. The abstract has been revised to improve clarity and conciseness by removing redundant statements and restructuring it into a point-focused summary. The revised version emphasizes key contributions, methodology, and validated results in a compact form, in line with the journal’s expectations

**• The quality of the figures is very poor. They must be updated with the HD quality resolution**

We thank the reviewer for this comment. We have improved the quality of the figure in the revised manuscript.

**• The introduction portion is relatively short. The following articles shall be included to strengthen the same.**

**o** [**https://doi.org/10.1155/mmce/9118129**](https://doi.org/10.1155/mmce/9118129) **o** [**https://doi.org/10.1016/j.aeue.2025.156184**](https://doi.org/10.1016/j.aeue.2025.156184)**.**

We thank the reviewer for this valuable suggestion. Relevant and recent references have been cited in the revised manuscript.

**• The research gap should be identified and included in the introduction. Also, the key objectives/novelties shall be reported in the introduction.**

We thank the reviewer for this important comment. In the revised version, we have clearly identified the limitations of existing implantable CP antennas—such as narrow axial ratio bandwidth (ARBW), high SAR, larger size, and limited gain—and formulated the research gap accordingly

**• The authors have mentioned the Methods for designing & testing the antenna in Figure 1, which is self-understood. It shall not be part of the research paper.**

The fig. 1 contains the final design and its parameter values. The unnecessary information is removed.

**• There should be a preamble before any topic/subtopic. This shall be maintained throughout the paper**

We thank the reviewer for this valuable suggestion. We agree that brief introductory (preamble) statements before each major section and subsection improve readability and logical flow. Accordingly, in the revised manuscript, concise preambles have been added at the beginning of all sections

**• The authors have included the bending analysis. Could the authors include how much the maximum bending shall be applied to maintain the desired response.**

We thank the reviewer for the valuable comment. We would like to clarify that the manuscript does not provide bending analysis. The design and performance analysis of the antenna under the planar form.

**• The manuscript should be revised with appropriate English grammar**

We thank the reviewer for this comment. The manuscript has been carefully revised to improve language and readability.
